# Some Points from Recent Circulars

**Published:** 1915-09-18

**Authors:** 


### NATIONAL INSURANCE ACT DEVELOPMENTS.

### Some Points from Recent Circulars.

Since the commencement of the war the Insurance Commissioners have issued many special instructions relating to the administrative difficulties in connection with National Insurance to which the war has given rise. It might, perhaps, have been thought that by this time all the special problems would have been dealt with, but experience has shown that new difficulties are constantly arising, and, further, that as the circle of those directly affected by the war is extended it is necessary from time to time to repeat announcements, in slightly revised form, for the benefit of those who have become affected since the original announcements were made.

An illustration is afforded in the circular recently issued (A.S. 172) which deals with the continuity of insurance of persons engaged in temporary non-insurable employment in connection with the war.

The Commissioners have received inquiries from various sources as to the position in insurance of persons, normally insured under the National Insurance Acts, who are temporarily engaged, whether abroad or in the United Kingdom, in non-insurable civilian occupations in connection with the war, and the circular to which reference has been made is designed to furnish information to those persons who are affected.

Among the cases cited are (*a*) nurses to His Majesty's Forces employed by the Army Council at home, or abroad, for the period of the war, or shorter period; and (*b*) nurses, orderlies, etc., employed abroad by the British Red Cross Society, or other similar organisation.

### Temporary Non-Insurable Employment.

The general position brought out by the circular is that if the approved society of which the insured person is a member is satisfied that the change of occupation is of a purely temporary character, and limited to the period of the war, the member must be treated as temporarily unemployed. This means that the member would really be treated as continuing in insurance, although for the time being contributions would not be payable. While resident in the United Kingdom the member would be entitled to the ordinary benefits, but if, and while, the member is abroad, no benefits (except maternity benefit) would be payable. In the ordinary way the position of such a member would be reviewed by the approved society on the expiration of twelve months from the date of the commencement of the "temporary unemployment." The Commissioners proceed in the circular to notify approved societies that when, upon review of the circumstances of a case at the end of the period of twelve months, they are satisfied that there has been no permanent change of normal occupation, the privilege of continuing in insurance is to be granted to the member.

### How Nurses are Affected.

"In the particular case of nurses employed temporarily by the Army Council for service during the war, including nurses of the Territorial Force Nursing Service, special arrangements have been made with the Army Council to whom a certificate of exception from insurance has been granted. Under the terms of this certificate the Army Council have undertaken to pay the arrears of those nurses who were insured persons at the time of entering on their Army employment, and arrangements have been made for the nurse to be supplied with a stamped arrears card, and a specially printed cover in which to forward it to her society.

"This applies only to nurses employed by the Army Council. Nurses employed by the British Red Cross Society, or other similar organisation, are, while resident in the United Kingdom, compulsorily insured (subject to the usual conditions), and contributions will be paid in the ordinary manner in respect of any weeks of employment."

### New System of Collection of Contributions from Army Members.

Another very important circular, dated August 1915, shows that the Insurance Commissioners are fully aware of the difficulties that have arisen through the stamping of the special "Army" contribution cards and the collection of those cards by the approved societies. The circular (257 A./A.G.D.) states that "the experience gained since the outbreak of the war has convinced the Commissioners that the present method of collecting the contributions of soldiers by means of stamps and cards is in practice unsatisfactory, very many cards failing to reach the societies entitled to them, and they have accordingly arranged with the Army Council that the stamping of Army cards shall cease as from the beginning of the present half-year. In future the contributions of soldiers will be paid to the Commissioners in cash, but no claim for credit for the contributions of an Army member will be made by the society during his period of service except in the case of transfers out of the society. In the normal case, the contributions from July 5, 1915, or from enlistment, if after that date, will be claimed on the member's discharge or death. Arrangements will, however, be made for giving provisional credit half-yearly on the estimated Army membership of the Society."

The circular then proceeds to describe in detail, for the benefit of officials of approved societies, the exact procedure to be adopted. It is quite unnecessary for us to comment on these details, which appear to be quite straightforward and practicable, but, with regard to the general plan of the alteration, we feel that it is only just to remark that it is undoubtedly a great improvement upon the "card-stamping" system.

